# Out-of-hours C-reactive protein point-of-care testing and its association with long-term healthcare-seeking

**DOI:** 10.1080/02813432.2026.2706193

**Published:** 2026-08-13

**Authors:** Jesper Lykkegaard, Jonas K. Olsen, Claus Høstrup Vestergaard, Malene Plejdrup Hansen, Linda Huibers

**Affiliations:** ^a^ Research Unit of General Practice, Department of Public Health, University of Southern Denmark, Denmark; ^b^ Research Unit for General Practice, Aarhus, Denmark; ^c^ Center for General Practice (CAM AAU), Aalborg University, Denmark; ^d^ Department of Public Health, Aarhus University, Aarhus, Denmark

**Keywords:** Point-of-care testing, C-reactive protein, after-hours care, health services utilization, primary health care

## Abstract

**Background:**

Increased diagnostic testing may contribute to clinically unnecessary healthcare-seeking.

**Aim:**

To analyse whether patients exposed to general practitioners (GPs) who frequently use C-reactive protein point-of-care testing (CRP-POCT) have different long-term use of out-of-hours (OOH) primary care services.

**Design and setting:**

Register-based time-to-event analysis of OOH primary care in the Central Denmark Region (population 1.3 million). The service functions as gatekeeper to hospital care. The GPs work a limited number of shifts per month.

**Methods:**

All in-person clinic consultations at the OOH clinics from 2014 to 2017 were included. The exposure was the consulted GP’s adjusted tendency to perform CRP-POCT relative to peer GPs, based on the latest 50 consultations. Covariates included patient characteristics, GP activity level and experience in OOH care, and time of consultation. Hazard ratios (HRs) for revisiting OOH primary care were estimated.

**Results:**

A total of 862,733 consultations were included. The proportion of consultations involving CRP-POCT increased from 12% in 2014 to 19% in 2017. Kaplan–Meier estimates showed that it took 2.5 years before half of the patients had returned for an OOH consultation. The adjusted HR for revisiting was 1.038 (95% CI = 1.023 to 1.054) for patients seen by a GP with twice the average CRP-POCT use or higher, compared with those seen by a GP with average use.

**Conclusion:**

Being seen by a GP with a high propensity to use CRP-POCT was associated with increased long-term OOH all-cause healthcare-seeking. This may reflect effects of CRP-POCT itself, correlated GP characteristics, or both.

## HOW THIS FITS IN

A prior controlled study found that patients who received a C-reactive protein point-of-care test (CRP-POCT) were more likely to express the intention to revisit if experiencing similar symptoms, compared to those who did not receive the test. This study found that, following a single out-of-hours primary care consultation, patients who happened to be seen by a general practitioner (GP) who frequently uses CRP-POCT had a small but statistically significant and long-lasting increase in the likelihood of future out-of-hours consultations, compared to those seen by a GP with average test use. The findings suggest that the growing use of CRP-POCT may increase demand for out-of-hours primary care.

## Introduction

Healthcare-seeking behaviour is a major driver of resource use in healthcare systems. While clinical needs influence demand, healthcare utilisation is also shaped by supply-side factors, such as diagnostic testing, which may encourage patients to use of services [1].

C-reactive protein point-of-care test (CRP-POCT) is a rapid diagnostic tool used in primary care to measure capillary blood levels of C-reactive protein, a non-specific marker of inflammation. In recent years, CRP-POCTs have become increasingly used in out-of-hours (OOH) primary care, often forming part of brief, one-off consultations with unfamiliar general practitioners (GPs) [2–5]. The test is primarily recommended to support decision-making and antibiotic stewardship in patients with acute respiratory tract infections, particularly for ruling out serious conditions such as pneumonia [6–9]. However, studies indicate that readily available CRP-POCTs are also used for a broad spectrum of other types of diagnostic uncertainties, including gastrointestinal, urinary, and skin symptoms as well as general malaise [4,10,11].

Beyond its clinical impact, the availability of CRP-POCTs may influence both GP behaviour and patient expectations. In the OOH primary care services in Denmark, CRP-POCTs are readily accessible, often positioned within arm’s reach of the GP [12]. This ease of access may increase the likelihood that testing is performed, even when clinical indications are unclear. Patients exposed to such testing may come to view it as an essential component of care, potentially increasing the likelihood of revisiting OOH services for similar concerns in the future [13–15]. This hypothesis is supported by the finding that patients who got a CRP-POCT in a randomized trial expressed a higher level of intention to revisit in case of similar symptoms [16].

At the Danish OOH primary care clinics, patients are assigned to the next available GP, who typically works only a few shifts per month. Like findings from Norway, Danish GPs vary substantially in their use of CRP-POCTs, thereby exposing patients to different likelihoods of being tested [4,12]. This variation within a highly standardised service model offers a unique opportunity to study associations between test use and subsequent patient behaviour. Thus, this study aimed to analyse the association between the level of CRP-POCT use by the general practitioner consulted and patients’ long-term use of out-of-hours primary care services.

## Methods

### Design and population

We conducted a register-based time-to-event cohort study. The cohort included all patients who had an in-person clinic consultation at the OOH primary care in the Central Denmark Region between 1 January 2014 and 31 December 2017. Patients were followed from the date of the index consultation until their next in-person clinic consultation with the OOH service or the end of follow-up. The study is reported in accordance with the Strengthening the Reporting of Observational Studies in Epidemiology (STROBE) guidelines [17].

### Setting

The Central Denmark Region has a population of approximately 1.3 million citizens, all of whom are covered by the OOH primary care service, operated jointly by the region’s GPs. The OOH service functions as a mandatory gatekeeper to secondary care. Patients are required to contact the service by telephone prior to receiving care. All calls are answered by GPs. Patients are managed over the phone or referred to an in-person clinic consultation, a home visit, or direct referral to hospital. If follow-up care is deemed necessary after an OOH consultation, the patient is advised to consult their regular daytime GP. During the study period, the OOH service was available from 16:00 to 08:00 on weekdays, and 24 h a day during weekends and public holidays [18]. Today nurses often perform CPR-POCT before the patient is assigned to a GP, but in 2017, it was generally the consulted GP who decided whether to test [19].

All the healthcare services are tax-funded [20]. GPs working OOH shifts are remunerated on a fee-for-service basis. Performing a CRP-POCT is reimbursed through a specific remuneration code (7120), with a fee of approximately £8 (9.5 EUR), in addition to the standard consultation fee.

### Data sources and variable definitions

Data were retrieved from the regional OOH registration system at the contact level. For each in-person clinic consultation, the following information was extracted: date and time; the patient’s unique personal identification number, age, and sex; the GP’s authorisation number; and whether remuneration was claimed for a CRP-POCT. Information on the patients’ daytime GP contacts was obtained from the National Health Insurance Service Register, and dates of death and emigration from the Danish Civil Registration System.

Patients were characterised by sex, age, and the number of general practice contacts (both daytime and OOH) in the preceding 12 months, used as a proxy for overall healthcare utilisation and need. GPs were characterised by the number of OOH shifts worked in the preceding 180 days, used as a proxy for recent experience in OOH care, and the GP’s number of patients seen in the preceding hour, used as a proxy for activity level at the time of the consultation. These variables could not be calculated if the consultation occurred within the first 180 days of the study period (for experience) or within the first hour of a shift (for activity level).

Each consultation was further characterised by the number of hours until the patient’s own daytime GP service reopened, and the calendar year and month.

### Analyses

For each in-person clinic consultation, the exposure variable was the consulted GP’s adjusted level of CRP-POCT use relative to GP peers, based on the GP’s previous 50 OOH consultations. To estimate the use, we constructed a binomial regression model adjusted for patient, GP, and consultation characteristics. The model allowed for the prediction of the expected number of CRP-POCTs per GP. To ensure robust estimates, GPs with fewer than two OOH shifts per year on average were excluded. Where necessary, missing data for covariates were handled by introducing a separate missing category.

To investigate the association between the consulted GP’s CRP-POCT use and the patient’s time to next OOH in-person clinic consultation (revisiting), we performed a Fine and Gray competing risk regression. The exposure variable was modelled using restricted cubic splines with four knots to allow for non-linear effects [21].

Patients were included at each OOH in-person clinic consultation and followed until their next in-person clinic consultation with the OOH services, death, emigration, or the end of the study period. Death and emigration were treated as competing events.

Results are reported as hazard ratios (HRs) with 95% confidence intervals (95% CI). All regression models included robust standard errors clustered at the GP level to account for repeated measurements. All statistical analyses were conducted using Stata version 18 (StataCorp LLC, College Station, TX, USA).

## Results

**Figure 1.**
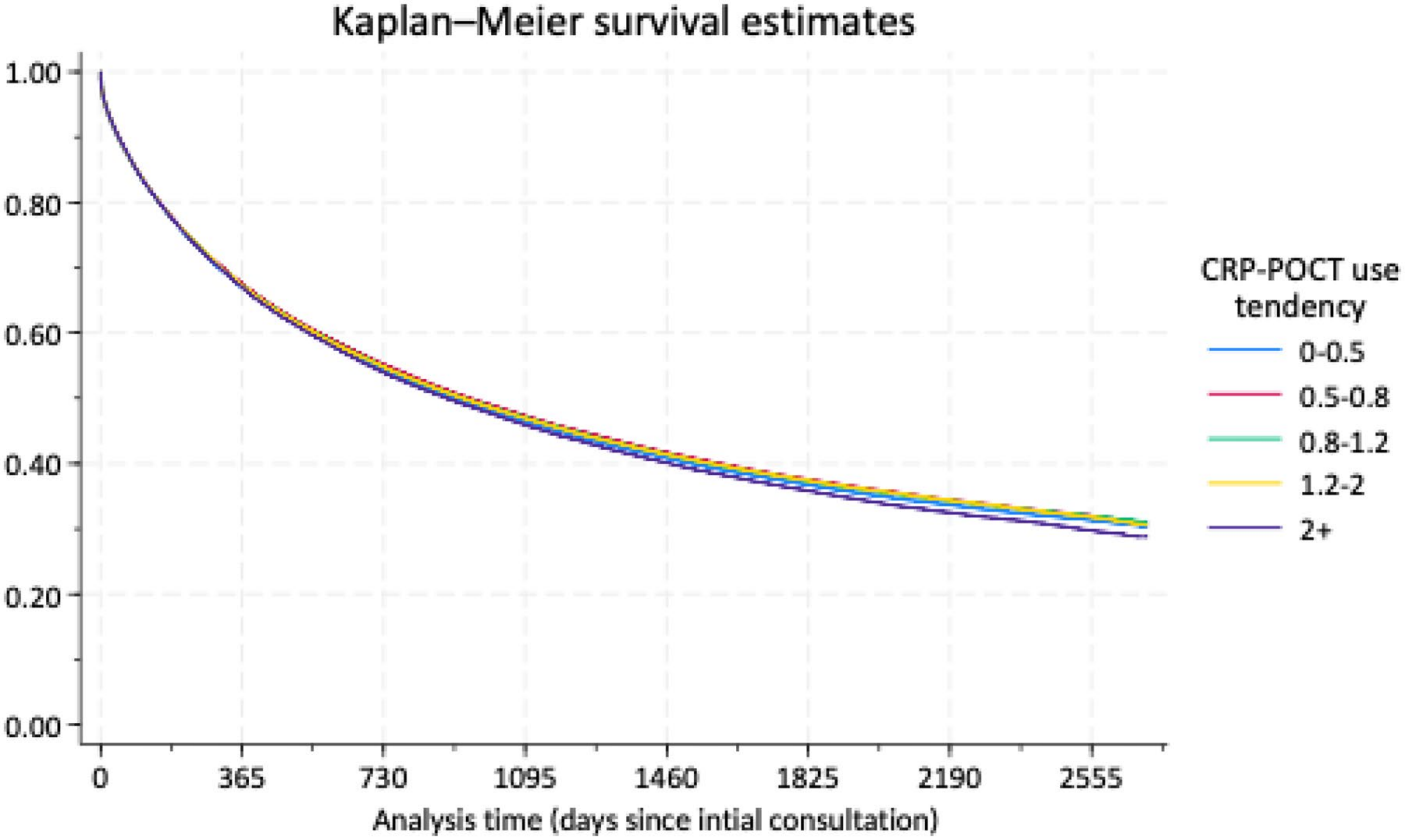
Kaplan–Meier curves showing time to first revisit to OOH primary care according to the consulted GP’s adjusted CRP-POCT use relative to peer GPs.

During the study period, 971 GPs worked in the out-of-hours primary care services, and 488,459 patients consulted them; the study was based on 862,773 in-person OOH consultations. The proportion of consultations in which a CRP-POCT was performed increased annually, from 12% in 2014 to 19% in 2017. Kaplan–Meier estimates showed that it took 2.5 years before half of the patients had revisited the OOH primary care (Figure 1).

Table 1 presents the factors associated with a CRP-POCT being performed during an OOH consultation. The positively associated factors included the patient having high prior contact frequency in both daytime general practice and OOH primary care, the GP having worked many OOH shifts in the preceding 180 days, the GP having a lower patient load in the hour preceding the consultation, and the consultation occurring at a time more than three hours before the next opening hours of the patient’s daytime GP practice. CRP-POCT was also more likely performed for female patients, with increased patient age up until 60–69 years, and when the consultation occurred later in the study period or during the winter season (Supplementary Table 1S).

**Table 1.** Risk ratios of CRP-POCT in 862,733 OOH primary care consultations in the Central Denmark Region (2014–2017).

Consultation characteristics		Risk ratio adjusted	95% Confidence interval
The patient’s number of GP daytime- and OOH consultations during the past year	0–2	1 (reference)	
	3–5	1.23	1.20-1.25
	6–9	1.39	1.36-142
	10–19	1.56	1.53-1.59
	20+	1.72	1.68-1.75
The GP’s number of OOH shifts during the past 180 days	0–2	1 (reference)	
	3–4	1.03	1.00-1.05
	5–10	1.05	1.03-1.07
	11+	1.14	1.11-1.17
The GP’s number of patients treated during the past hour	0–4	1 (reference)	
	5–10	1.00	0.99-1.02
	11+	0.86	0.83-0.89
The number of hours until GP regular daytime opening hours	0–3	1 (reference)	
	4–15	1.13	1.06-1.21
	16–47	1.14	1.07-1.22
	48+	1.06	1.00-1.13

The risk ratios are adjusted for the above variables plus patient sex, age (categorized <2 years, 3–9, 10–19, and in the above 10-year-intervals), calendar year (2014–2017), and month. Abbreviations: CRP-POCT, C-reactive protein point-of-care test; OOH, Out-of-hours; GP, general practitioner. See the full output of the analysis in supplementary Table 1S.

The consulted GPs’ adjusted CRP-POCT use at the time of consultation varied considerably. In 21% of consultations, the GP’s use was less than half of the expected level, while in 6% it was twice the expected or higher (Table 2). For 5% of consultations, the use could not be estimated because the GP had conducted fewer than 50 prior OOH consultations.

**Table 2.** Association between consulted GP’s level of CRP-POCT use and patients’ four-year hazard ratio of revisiting OOH primary care in the Central Denmark Region.

	Relative to expected	Share of the consultations	Hazard ratio	95% confidence interval
CRP-POCT use	−0.49	21%	1.003	0.994-1.013
	0.50-0.79	18%	0.986	0.976-0.996
	0.80-1.19	23%	1 (reference)	
	1.20-1.99	25%	0.992	0.983-1.003
	2.00-	6%	1.038	1.024-1.053
	NA	5%	–	–

Abbreviations: GP, general practitioner; CRP-POCT, C-reactive protein point-of-care test; OOH, Out-of-hours; NA, not applicable. The CRP-POCT use was estimated based on the GP’s latest 50 consultations and relative to expected given the patients’ sex, age, and prior frequency of consulting, the GP’s experience working the OOH primary care services and activity level at the hour of the consultation, and time of the consultation (year, month, and hours until GP daytime opening hours).

Compared with patients who were seen by GPs with CRP-POCT use ranging from 80% to 120% of the expected, patients seen by GPs with twice the expected or higher had a significantly higher rate of revisiting (HR = 1.038; 95% CI = 1.023 to 1.054) and patients seen by GPs with lower use between 50–79% had a lower HR of revisiting. A clear dose-response relation between CRP-POCT use and revisiting was not found (Table 2).

The Kaplan–Meier curves indicated that the association between consulted GPs’ CRP-POCT use and patients’ future revisits to OOH services persisted throughout the four-year study period (Figure 1).

## Discussion

### Summary

This register-based cohort study found substantial variation in the use of CRP-POCT among GPs working shifts in the OOH primary care in the Central Region of Denmark. It took 2.5 years before half of the patients had revisited the OOH primary care service. Being seen by a GP with a CRP-POCT use twice the expected level or higher was associated with a small (4%) but long-lasting increase in the likelihood of revisiting the OOH services.

### Strengths and limitations

The strengths of this study include use of highly valid and virtually complete remuneration data. Follow-up was further strengthened by the fact that the included service constituted the only OOH primary care provider in the region throughout the study period. Additionally, the service allocated patients to GPs in a manner largely determined by chance, resulting in exposure to clinicians with substantially different use of CRP-POCT, although without true randomization. We restricted the study period to 2014–2017, since as of around 2018 nurses began to perform CRP-POCT prior to the GPs’ assessment of the patients [19].

Regarding potential limitations, key issues to consider include the plausibility of a true long-term effect of CRP-POCT on allcause OOH helathcare-seeking, the suitability of the study design for assessing the association, and whether a HR of 1.038 observed in 6% of consultations represents a clinically meaningful effect size.

Long-term associations between CRP-POCT use and healthcare seeking have not been investigated before. However, research shows that CRP-POCT can be associated with increased short-term healthcare-seeking with the regular GP e.g. for retesting [22]. When a GP interprets a CRP-POCT result as supporting the diagnosis of a viral infection, the patient may feel reassured and as such less likely to seek healthcare for similar symptoms in the future [23]. However, our findings could indicate the opposite that patients come to perceive the CRP-POCT as a routine or essential part of the consultation, potentially fostering the belief that testing is necessary for appropriate care, which increases future healthcare-seeking. This hypothesis is supported by Cals et al.’s finding in a controlled trial that performing CRP-POCTs increased patients’ stated intention to seek care for similar symptoms in the future [16].

The exposure in our study design, a 5–10-minute consultation, may be considered insufficient to exert a lasting influence on subsequent service use. However, as patients’ most recent encounter with out-of-hours care before revisiting, it likely served as their main source of information, informing both expectations of the service and judgments about when to seek care again.

The observed variation in the GPs’ CRP-POCT use extends beyond what would be expected by chance. Based on fifty consultations per GP and random distribution of the CRP-POCTs, around two in a thousand consultations would have been with a GP having a current CRP-POCT use more than twice the observed 16% average. This is much lower than the observed 6% of consultations. An alternative explanation, beyond differences in GPs’ individual tendencies to use CRP-POCT, is variation in patient case-mix. For example, a higher proportion of patients presenting with pneumonia-like symptoms may lead to increased use of CRP-POCT. Such variation is likely during the winter peak of respiratory infections and may also vary by weekday and time of day. To account for this, expected CRP-POCT use rates were estimated according to month, weekday, and proximity to regular general practice opening hours, and high users were defined as GPs with observed use rates at least twice their expected rate. Further, expected rates were according to the age, sex, and prior healthcare seeking of the 50 patients. Nevertheless, residual confounding may remain, and information on reasons for encounter, symptoms, clinical findings, and diagnoses would have improved the study. Other studies agree to the existence of substantial variation in GPs’ CRP-POCT use regardless of patient case-mix [4,22,24].

We did not compare revisiting rates between patients who received CRP-POCT and those who did not, as such analyses would be highly susceptible to confounding by indication. Patients selected for CRP-POCT are likely to differ systematically, as they present with symptoms warranting testing and may have a higher underlying propensity to reconsult, both due to clinical condition and healthcare-seeking behaviour. In addition, previous studies suggest that CRP-POCT is associated with increased short-term revisiting, further complicating interpretation [22].

Other GP characteristics may act as confounders if they are associated with both the propensity to use CRP-POCT and patients’ likelihood of revisiting. For example, some GPs appear to be more activity-prone, characterised by a higher use of diagnostic tests and interventions [24]. Such practice styles may both increase the use of CRP-POCT and influence patients’ subsequent healthcare-seeking behaviour, for instance by reinforcing the perception that medical evaluation is warranted. We were unable to assess this directly. However, Smits et al. reported that patients registered with practices characterised by higher use of diagnostic and therapeutic procedures had increased contact rates with out-of-hours primary care, supporting this potential mechanism [25]. Consequently, the observed association may reflect the effects of CRP-POCT itself, correlated GP characteristics, or a combination of both.

Only 6% of consultations in our study involved a GP with a CRP-POCT use associated with the highest risk of revisiting (HR: 1.038). This corresponds to a relatively small proportion of consultations that might be preventable through reductions in CRP-POCT testing. At the individual patient level, the observed increase in risk is likely of limited clinical relevance. However, out-of-hours (OOH) primary care represents the main entry point to the broader OOH healthcare system, and even small effects may therefore be meaningful at the system level. This is particularly relevant given that the average use of CRP-POCT in Danish and Norwegian OOH primary care has approximately doubled since our study period [2,5]. Furthermore, comprehensive interventions aimed at reducing OOH healthcare-seeking or strengthening OOH telephone triage have not demonstrated measurable effects on overall healthcare-seeking [26,27], despite requiring considerable resources and system-level changes. In this context, a 3.8% increase suggests that shifts in CRP-POCT use may contribute to unintended increases in healthcare utilization at the population level to an extent otherwise difficult to bring about.

Although a clearer dose–response relationship would have strengthened confidence that the observed association is not due to chance, our analysis was based on a preplanned hypothesis test and yielded a statistically significant, confirmatory result.

We included only in-person clinic consultations, both as index contacts and as revisits. As all patients must initially contact OOH primary care by telephone, the triaging GP determines whether the patient is referred to a clinic consultation or managed with telephone advice. Consequently, our data do not capture the overall rate of patient recontact with OOH primary care services. However, in-person clinic consultations are the most resource-intensive type of contact and were therefore selected as the outcome. We cannot tell if our result means that patients who saw a high user of CRP-POCT recontacted sooner or were more often booked for a consultation the next time they called the OOH primary care service.

### Comparison to other studies

Recent studies from Scandinavian settings have raised concerns about the overuse of CRP-POCT; specifically, its frequent use prior to clinical assessment, its application in patients without clear indication, and its growing status as a routine rather than selective diagnostic tool [4,12,19,28]. A recent study found a positive relationship between level of CRP-POCT and antibiotic prescribing, suggesting that at some point, more CRP-POCTs do not contribute to more prudent antibiotic prescribing [12].

### Implications for research and/or practice

CRP-POCTs are used extensively in OOH primary care in countries such as Denmark, the Netherlands, Norway, Sweden, and Switzerland [2,4,29,30], but remain far less commonly used in the UK [31]. Globally, broader implementation of CRP-POCT is considered an important tool in efforts to reduce unnecessary antibiotic use and thereby combat antimicrobial resistance [32]. However, under- and overuse needs to be balanced. While most countries could benefit from introducing CRP-POCT in OOH primary care settings, countries with very high utilisation, such as the Scandinavian countries, may focus on reducing inappropriate use. In addition to potentially increasing clinically unwarranted healthcare-seeking behaviour, indiscriminate use of CRP-POCT may contribute to unnecessary antibiotic prescribing, as testing patients with a low pre-test probability of bacterial infection reduces the positive predictive value of elevated CRP results [12].

All healthcare systems should aim to follow evidence-based guidelines and use CRP-POCT only when clinically indicated, based on history taking and physical examination [32]. Routine or automatic use of CRP-POCT in OOH settings, without clinical justification, should be avoided.

Future research might concentrate on GPs with a high use of CRP-POCT to gain more insights into their consultation processes and decision-making. Furthermore, research should focus on defining the accurate use of CRP-POCT, as one may expect an upper boundary for relevant test use.

## Conclusion

We found a GP variation in the use of CRP-POCT in OOH primary care. Patients who had an in-person clinic consultation with a GP among those with the highest use had a slightly higher likelihood of revisiting OOH primary care during the next 4 years. We do not know the relevance of the revisits. High GP use of CRP-POCT could be a proxy for other GP characteristics that somehow impacted the patients’ healthcare-seeking behaviour, such as in general being more activity-prone.

## Supplementary Material

Supplemental Material

## Data Availability

Data supporting the findings of this study was used under a license granted specifically for the current study and therefore is not publicly available according to the data protection regulations of the Danish Data Protection Agency, Statistics Denmark and the Danish Health and Medicines Authority.
