# Supplementary material for: Out-of-hours C-reactive protein point-of-care testing and its association with long-term healthcare-seeking
Source: Scand J Prim Health Care. 2026 Aug 13;44(1):2706193. doi: 10.1080/02813432.2026.2706193 (PMC13479521; doi:10.1080/02813432.2026.2706193)
Supplement: Supplementary file 1 [file IPRI_A_2706193_SM3711.docx]

| **Table 1S: Risk ratios of CRP-POCT in 862,733 OOH primary care consultations in the Central Denmark Region (2014-2017) – full regression analysis report.** | | | |
| --- | --- | --- | --- |
| Consultation characteristics: | | Risk ratio adjusted | 95% Confidence interval |
| Patient’s sex | Female | 1 (reference) |  |
|  | Male | 0.89 | 0.88-0.90 |
| Patient’s age *(in years)* | 0-9 | 1 (reference) |  |
|  | 10-19 | 1.02 | 1.00-1.05 |
|  | 20-29 | 1.18 | 1.16-1.21 |
|  | 30-39 | 1.23 | 1.20-1.26 |
|  | 40-49 | 1.28 | 1.25-1.31 |
|  | 50-59 | 1.29 | 1.26-1.32 |
|  | 60-69 | 1.31 | 1.27-1.34 |
|  | 70-79 | 1.29 | 1.25-1.32 |
|  | 80-89 | 1.22 | 1.18-1.27 |
|  | 90+ | 1.05 | 0.98-1.13 |
| Calendar year | 2014 | 1 (reference) |  |
|  | 2015 | 1.19 | 1.17-1.21 |
|  | 2016 | 1.37 | 1.34-1.39 |
|  | 2017 | 1.48 | 1.46-1.51 |
| Month | January | 1 (reference) |  |
|  | February | 1.11 | 1.08-1.14 |
|  | March | 1.02 | 0.99-1.05 |
|  | April | 0.88 | 0.86-0.91 |
|  | May | 0.80 | 0.78-0.83 |
|  | June | 0.77 | 0.74-0.79 |
|  | Juli | 0.81 | 0.79-0.84 |
|  | August | 0.80 | 0.78-0.83 |
|  | September | 0.86 | 0.83-0.89 |
|  | November | 1.00 | 0.97-1.02 |
|  | December | 1.07 | 1.04-1.10 |
| The patient’s number of GP daytime- and OOH consultations during the past year | 0-2 | 1 (reference) |  |
|  | 3-5 | 1.23 | 1.20-1.25 |
|  | 6-9 | 1.39 | 1.36-142 |
|  | 10-19 | 1.56 | 1.53-1.59 |
|  | 20+ | 1.72 | 1.68-1.75 |
| The GP’s number of OOH shifts during the past 180 days | 0-2 | 1 (reference) |  |
|  | 3-4 | 1.03 | 1.00-1.05 |
|  | 5-10 | 1.05 | 1.03-1.07 |
|  | 11+ | 1.14 | 1.11-1.17 |
| The GP’s number of patients treated during the past hour | 0-4 | 1 (reference) |  |
|  | 5-10 | 1.00 | 0.99-1.02 |
|  | 11+ | 0.86 | 0.83-0.89 |
| The number of hours until GP regular daytime opening hours | 0-3 | 1 (reference) |  |
|  | 4-15 | 1.13 | 1.06-1.21 |
|  | 16-47 | 1.14 | 1.07-1.22 |
|  | 48+ | 1.06 | 1.00-1.13 |
| The risk ratios are adjusted for the above variables plus patient sex, age (categorized <2 years, 3-9, 10-19 and in the above 10-year-intervals), calendar year (2014-2017), and month. Abbreviations: GP, general practitioner; CRP-POCT, c-reactive protein point-of-care test; OOH, Out-of-hours. | | | |
